# Trajectories of kinesiophobia over the first six months after cardiac pacemaker or defibrillator implantation: clinical, psychological, and rehabilitation predictors and their relation to functional capacity

**DOI:** 10.3389/fcvm.2026.1939242

**Published:** 2026-10-07

**Authors:** Chunli Zhang, Xiaoping Guo

**Affiliations:** Department of Cardiology, The 980th Hospital of the Joint Logistic Support Force of the Chinese People’s Liberation Army (Bethune International Peace Hospital), Shijiazhuang, Hebei, China

**Keywords:** anxiety, cardiac pacemaker, cardiac rehabilitation, depression, implantable cardioverter defibrillator, kinesiophobia, self-efficacy, trajectory analysis

## Abstract

**Objective:**

Kinesiophobia, an excessive fear of physical movement, is common after implantation of a cardiac pacemaker or implantable cardioverter defibrillator and may limit rehabilitation and recovery. Because fear of movement changes over time, a single measurement cannot capture how it evolves. We aimed to identify distinct trajectories of kinesiophobia during the first six months after implantation, to relate them to functional capacity, and to identify baseline factors that predict an unfavourable course.

**Methods:**

In a single centre retrospective cohort based on routinely collected structured follow-up data, 160 consecutive patients undergoing first implantation of a pacemaker or defibrillator were assessed 24 to 48 h after the procedure and again at 1, 3, and 6 months. Kinesiophobia was measured with the Tampa Scale for Kinesiophobia Heart and functional capacity with the six-minute walk distance. Group-based trajectory modelling identified latent trajectories. Baseline characteristics were compared across classes, and multinomial logistic regression identified independent predictors of class membership.

**Results:**

Mean kinesiophobia fell from 42.9 to 35.2 over six months, and the proportion above the high-fear threshold fell from 76.2% to 37.5%. Three trajectories emerged: low and declining (28.8%), moderate and declining (45.0%), and high and persistent (26.2%). The persistent group stayed above the clinical threshold throughout and walked the shortest distance at every visit. Compared with the low group, independent predictors of a persistent course were higher depression (odds ratio 2.26), higher anxiety (1.68), lower exercise self-efficacy (0.89), shorter baseline walk distance (0.55 per 10 m), and absence of exercise guidance (0.04 for having received guidance).

**Conclusion:**

Kinesiophobia after device implantation follows three trajectories, separated mainly by the overall level of fear. About one quarter of patients remain persistently fearful and physically limited; they can be identified early by distress, low self-efficacy, poor functional capacity, and lack of exercise guidance. Early screening and targeted exercise-based rehabilitation are warranted.

## 1 Introduction

Cardiac implantable electronic devices, including permanent pacemakers and implantable cardioverter defibrillators, are a mainstay of treatment for symptomatic bradycardia, advanced conduction disease, heart failure, and patients at risk of sudden cardiac death. As populations age and indications widen, the number of people living with a device continues to grow (1). Implantation reliably corrects the underlying electrical problem, yet a good outcome depends on more than device function. Returning to an active daily life is central to recovery, and regular physical activity is a core component of secondary prevention in cardiovascular disease (2).

Many device recipients do not resume their previous level of activity. One common but often overlooked barrier is kinesiophobia, defined as an excessive, irrational, and debilitating fear that movement or physical exertion will cause harm (3). The concept was first described in chronic musculoskeletal pain and is well captured by the fear-avoidance model, in which a catastrophic interpretation of bodily sensations produces fear, fear leads to avoidance of activity, and avoidance causes physical deconditioning that in turn deepens the fear (4). In patients with heart disease the fear often centres on the belief that exertion will dislodge the device or a lead, trigger a shock, or provoke another cardiac event (5, 6).

Fear of movement is not a benign concern. In coronary populations it is associated with lower physical activity, poorer functional performance, and worse quality of life (5, 7). It reduces participation in exercise-based cardiac rehabilitation (8) and predicts failure to start rehabilitation after a cardiac admission (9), and it has been linked to poor adherence to activity recommendations, particularly in older patients (10). The problem is not limited to coronary disease; fear of movement is also common in heart failure and after cardiac surgery (11).

Patients with an implanted defibrillator carry a particular psychological burden. Anxiety and depression affect a substantial minority, with recent pooled estimates of 22.6% for anxiety and 15.4% for depression (12, 13), and fear of shocks together with post-traumatic stress are well recognised in this group (14). Physical activity may fall after implantation, especially in patients who received the device for secondary prevention (15). These features together make fear of movement a plausible and clinically important problem after device implantation, yet it has rarely been studied in this setting.

The early period after device implantation has features that may make fear of movement especially likely to take hold. Patients are commonly advised to limit movement of the arm on the side of the implant for several weeks so that the leads can settle, and this sensible short-term precaution can be misread as a sign that the heart or the device is fragile. The generator sits in a shallow pocket where it can be seen and felt, which keeps attention on the device and on the wound. For recipients of a defibrillator there is the added anticipation of a shock, which can attach fear to any rise in heart rate. Legitimate early restrictions and lasting avoidance can therefore blend into one another, and a patient who remains uncertain about what is safe may continue to limit activity well beyond the point at which caution is warranted.

Set against this, cardiac rehabilitation remains underused. Even where structured programmes exist, many eligible patients are never referred or do not attend (16), and device recipients are under-represented because of lingering caution about exercise in this group (17). Fear of movement is one reason patients decline or withdraw, so a fear that goes unrecognised at the start can quietly undermine the very treatment that would help most. A clearer picture of who remains fearful, and for how long, is a necessary step toward closing this gap and matching the intensity of support to the patients who need it.

Despite its importance, kinesiophobia after device implantation has mostly been examined at a single point in time. A single measurement yields one prevalence figure and conceals two clinically relevant features. First, fear of movement is dynamic. For many patients it is highest in the early period after the procedure and recedes as confidence returns, whereas for others it persists. Second, the patient population is heterogeneous, so a single average value may hide subgroups whose fear follows very different courses. Cross-sectional studies in cardiac patients have already shown that fear of movement separates into low, moderate, and high profiles rather than forming a single homogeneous group (18, 19). What remains unknown is how these levels evolve over time after device implantation and, in particular, whether a subgroup exists whose fear does not resolve.

Group-based trajectory modelling offers a principled way to uncover such latent subgroups. It groups individuals who share a similar pattern of change over time without specifying the groups in advance (20). Because exercise-based cardiac rehabilitation improves functional capacity, quality of life, and clinical outcomes (21, 22), and dedicated programmes have been developed for device recipients, identifying patients whose fear is unlikely to resolve on its own would direct these resources to those who need them most. We therefore examined a six-month cohort with four structured assessments in patients receiving a pacemaker or defibrillator. The aims were threefold: to identify distinct trajectories of kinesiophobia over the first six months after implantation; to describe how these trajectories relate to an objective measure of functional capacity, namely the six-minute walk distance; and to identify the baseline clinical, psychological, and rehabilitation factors that independently predict an unfavourable trajectory, with attention to modifiable factors that could become targets for intervention.

## 2 Methods

### 2.1 Study design and setting

This was a single centre, retrospective, observational cohort study carried out in the Department of Cardiology of The 980th Hospital of the Joint Logistic Support Force of the Chinese People's Liberation Army (Bethune International Peace Hospital), Shijiazhuang, Hebei Province. The department runs a dedicated and fully staffed device follow-up service, which made complete structured assessment possible at every scheduled visit. These structured assessments, including the questionnaires and the six-minute walk test, are administered to all device recipients at fixed scheduled visits as part of the routine follow-up protocol, and the resulting records were retrieved retrospectively for the present analysis. Consecutive patients implanted between November 2023 and January 2026 were included. The study was approved by the Medical Ethics Committee of the 980th Hospital of the Joint Logistics Support Force of the Chinese People's Liberation Army (Approval No. 2023-KY-95) and was conducted in accordance with the Declaration of Helsinki. Because the study was a retrospective analysis of de-identified data collected during routine clinical care, the requirement for written informed consent was waived by the ethics committee.

### 2.2 Participants

Eligible patients were adults undergoing first implantation of a permanent pacemaker or implantable cardioverter defibrillator who were conscious, able to communicate, and whose records contained the structured assessments at all four scheduled visits. Patients were excluded if they had severe cognitive impairment or psychiatric illness that prevented valid self-report, a pre-existing musculoskeletal or neurological condition that independently limited mobility, a terminal comorbidity with life expectancy under six months, or an inability to perform the six-minute walk test for reasons unrelated to cardiac status. As a working benchmark, the target sample size was based on the convention of 5 to 10 participants for each item of the largest instrument, the 17-item Tampa Scale for Kinesiophobia Heart (23), which gave a minimum of 150; no formal power calculation was performed for the trajectory model or the regression analysis. In total, 160 patients met these criteria and were included. Inclusion was consecutive to limit selection bias, and a single experienced implanting team reduced procedural variation. Because the department provides the regional device follow-up service, patients returned to the same clinic for every scheduled check, which supported complete data collection across the six-month period.

### 2.3 Outcomes and assessment schedule

Assessments were performed at four time points: T0, 24 to 48 h after implantation, which served as baseline; T1 at 1 month; T2 at 3 months; and T3 at 6 months. Kinesiophobia was the primary outcome and functional capacity was a parallel outcome; both were measured at all four visits. Psychological and social variables, together with clinical and laboratory characteristics, were recorded at baseline and treated as predictors.

### 2.4 Measurements

### 2.4.1 Kinesiophobia

The Tampa Scale for Kinesiophobia, Heart version, a 17-item instrument validated for cardiac patients, was used to measure fear of movement. Total scores range from 17 to 68, with higher scores indicating greater fear; a score of 37 or above denotes a clinically high level of kinesiophobia (5, 6).

### 2.4.2 Anxiety and depression

Symptoms were assessed with the Hospital Anxiety and Depression Scale, which comprises an anxiety subscale and a depression subscale, each scored from 0 to 21, with higher scores indicating more severe symptoms (24, 25).

### 2.4.3 Exercise self-efficacy

Confidence in the ability to exercise was measured with the Self-Efficacy for Exercise scale, which ranges from 0 to 90, with higher scores indicating greater confidence (26, 27).

### 2.4.4 Perceived social support

Perceived support was measured with the Multidimensional Scale of Perceived Social Support, which ranges from 12 to 84, with higher scores indicating greater perceived support (28).

### 2.4.5 Functional capacity

The six-minute walk distance was measured along a flat, quiet corridor of fixed length following the American Thoracic Society guidelines, with standardised wording of encouragement and the same instructions at every visit (29). Patients rested before the test, and the distance walked in six minutes was recorded in metres. The test was supervised by staff trained in the protocol who could stop it for predefined safety reasons.

### 2.4.6 Clinical and rehabilitation variables

Baseline data comprised demographics (age, sex, body mass index, living arrangements, education, and income), New York Heart Association functional class, left ventricular ejection fraction, left atrial diameter, B-type natriuretic peptide, haemoglobin, estimated glomerular filtration rate, comorbidities, a symptom-burden score, device type, whether the patient had received structured exercise guidance, and self-reported regular exercise habit. Structured exercise guidance was defined as individualised, in-person instruction on safe physical activity delivered by a cardiologist or a trained cardiac nurse during the index admission, after implantation and before the baseline assessment. The instruction typically covered which movements of the arm on the implant side to avoid and for how long, a graded walking plan for the first weeks at home, symptom-based limits for exertion and, for defibrillator recipients, what to do in the event of a shock; most patients also received a written activity sheet. Brief, unstructured verbal advice of the kind given to all patients at discharge did not qualify. Receipt of guidance was recorded from the nursing documentation of the index admission.

### 2.5 Statistical analysis

Latent trajectories of kinesiophobia were estimated by group-based trajectory modelling, also termed latent-class growth modelling, using the lcmm package in R (20, 30). Time was coded in months (0, 1, 3, and 6). Within each class the trajectory was specified as a quadratic function of time, which accommodated the decelerating decline visible in the observed means and fitted better than a linear specification. In keeping with the latent-class growth approach, the model estimated class-specific fixed trajectories without within-class random effects; individual departures from the class trajectory are absorbed by the residual variance. Models with one to five classes were fitted, each with a grid search over multiple random starts to avoid local maxima. Model selection balanced the Bayesian information criterion and the Akaike information criterion, classification quality measured by entropy (with values above 0.8 indicating good separation), parsimony, clinical interpretability, and the requirement that no class contain fewer than 5% of patients. Each patient was then assigned to the trajectory with the highest posterior probability, and classes were ordered and labelled by their mean kinesiophobia level.

Baseline characteristics were compared across trajectory classes using one-way analysis of variance or the Kruskal–Wallis test for continuous variables, and the chi-square test or the Fisher exact test for categorical variables. Independent predictors of trajectory membership were examined with multinomial logistic regression, using the most favourable trajectory as the reference category. To keep the number of parameters compatible with the sample size, the model did not include the full set of baseline variables. Candidate predictors were those that differed across trajectories in the univariate comparisons, and where several of these captured overlapping constructs a single representative was retained: New York Heart Association class, natriuretic peptide, and the symptom score overlap with ejection fraction and walk distance as markers of cardiac severity and function, estimated glomerular filtration rate tracks age and haemoglobin, and regular exercise habit overlaps with guidance and self-efficacy, so these were not entered. The final model contained nine predictors, corresponding to 20 estimated parameters for 160 observations; keeping a single representative from each cluster of overlapping variables also limited collinearity among the predictors. Because this selection was informed by the observed data rather than by pre-specified hypotheses, the regression is reported as exploratory and hypothesis-generating. Continuous predictors were rescaled to clinically meaningful units to aid interpretation of odds ratios. Trajectories of the six-minute walk distance were summarised in parallel by class. A two-sided *P* value below 0.05 was considered statistically significant. Given the exploratory nature of the predictor analysis, no adjustment for multiplicity was applied and *P* values are reported descriptively. All analyses were performed in R.

Follow-up was complete, so no data were imputed for the trajectory analysis. For each candidate model the grid search used multiple random sets of starting values to reduce the risk of converging on a local maximum, and convergence was confirmed for the retained model. After classes were assigned, the adequacy of separation was checked using the average posterior probability of membership within each class, with values above 0.7 regarded as acceptable. Odds ratios from the multinomial model are reported for clinically meaningful increments: per 5 years of age, per 5% of ejection fraction, per 10 g/L of haemoglobin, per 10 m of baseline walk distance, and per single point of each psychological scale.

## 3 Results

### 3.1 Participant characteristics

A total of 160 patients were included, all with complete assessments at the four visits. The mean age was 71.7 ± 12.2 years, and 82 patients (51.2%) were women. Dual-chamber pacemakers predominated (145 patients, 90.6%), with smaller numbers of implantable cardioverter defibrillators (7 patients), single-chamber pacemakers (5 patients), and cardiac resynchronisation devices (3 patients). At baseline, mean kinesiophobia was 42.9 ± 7.7 and 122 patients (76.2%) scored at or above the high-fear threshold. Mean baseline values for anxiety, depression, exercise self-efficacy, and perceived social support were 8.4 ± 3.7, 6.6 ± 3.3, 51.3 ± 14.9, and 62.0 ± 10.5, respectively, and 90 patients (56.2%) had received structured exercise guidance. The cohort reflected a typical bradycardia-device population, in which most devices were dual-chamber pacemakers implanted for conduction disease, with a minority of defibrillators and resynchronisation devices. Comorbidity was common and many patients had limited functional capacity at baseline, consistent with the age and cardiac profile of the sample.

### 3.2 Trajectories of kinesiophobia

Across the whole cohort, mean kinesiophobia declined steadily from 42.9 ± 7.7 at baseline to 37.0 ± 8.1 at three months and 35.2 ± 8.4 at six months. The prevalence of high kinesiophobia fell from 76.2% to 70.0%, 51.9%, and 37.5% across the four visits. Group-based trajectory modelling supported a three-class solution (Table 1). The Bayesian information criterion fell steeply from the one-class to the three-class model and then flattened. The three-class model had high classification quality, with an entropy of 0.945, a Bayesian information criterion of 3,794.3, an Akaike information criterion of 3,757.4, and a log-likelihood of −1,866.7, and all three classes exceeded the minimum size. The average posterior probability of class membership was above 0.9 in every class, indicating that patients were assigned to their trajectory with little ambiguity. Four-class and five-class solutions produced small and clinically redundant groups with only marginal gains in the information criteria.

**Table 1:** Fit statistics for group-based trajectory models with one to five classes.

Classes (G)	Log-likelihood	AIC	BIC	Entropy	Smallest class, %
1	−2245.4	4,498.8	4,511.1	NA	100.0
2	−2037.3	4,090.6	4,115.2	0.888	46.3
3	−1866.7	3,757.4	3,794.3	0.945	26.3
4	−1806.8	3,645.7	3,694.9	0.935	20.6
5	−1783.5	3,607.0	3,668.5	0.926	11.3

The selected three-class model is shown in the third row. AIC, Akaike information criterion; BIC, Bayesian information criterion. Entropy ranges from 0 to 1, with higher values indicating better separation between classes; it is not defined for the one-class model (NA).

The three trajectories are shown in Figure 1. The low group (46 patients, 28.8%) began below the clinical threshold and declined further, from a mean of 33.3 to 26.3. The moderate group (72 patients, 45.0%), which was the largest, started above the threshold and improved steadily, crossing below it by three months (from 44.1 to 34.1). The persistent group (42 patients, 26.2%) started highest and showed only a slight decline, remaining well above the clinical threshold throughout follow-up (from 51.3 to 46.9).

**Figure 1:**
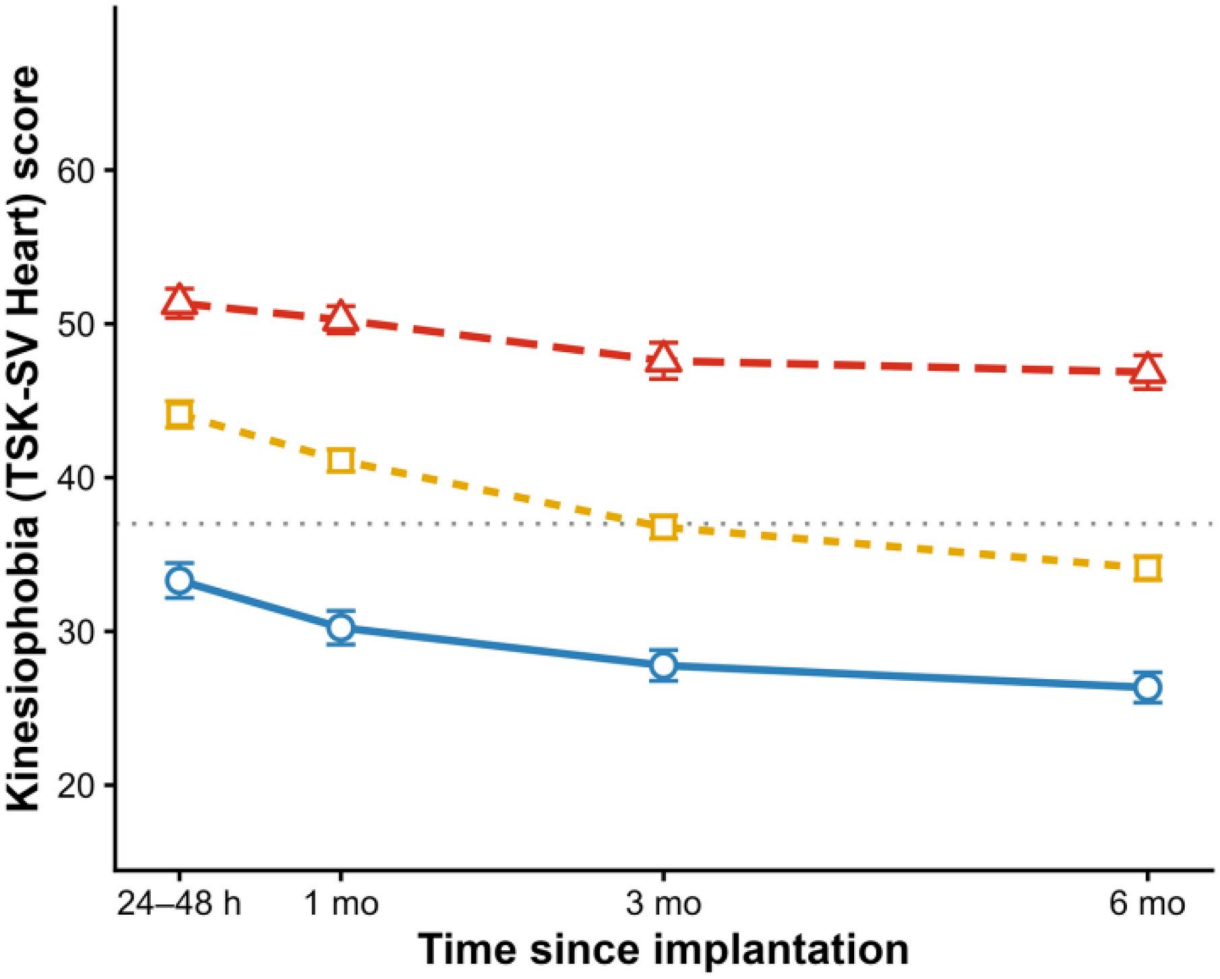
Trajectories of kinesiophobia over six months after device implantation. Mean Tampa Scale for Kinesiophobia Heart score with 95% confidence interval at 24 to 48 h, 1 month, 3 months, and 6 months for each latent trajectory class: low and declining (blue, solid line, circles), moderate and declining (gold, dashed line, squares), and high and persistent (red, dashed line, triangles). The dotted horizontal line marks the high-kinesiophobia threshold of 37.

### 3.3 Functional-capacity trajectories

The six-minute walk distance improved over time in all three groups but stayed clearly stratified by kinesiophobia trajectory throughout follow-up (Figure 2). The low group walked farthest at every assessment, from 392 to 479 m. The moderate group was intermediate, from 329 to 400 m. The persistent group walked least at every time point and showed the smallest absolute gain, from 251 to 295 m, a mean improvement of about 44 m compared with about 87 m in the low group and 71 m in the moderate group. The trajectory of greatest and most persistent fear therefore coincided with the poorest and least improving functional capacity.

**Figure 2:**
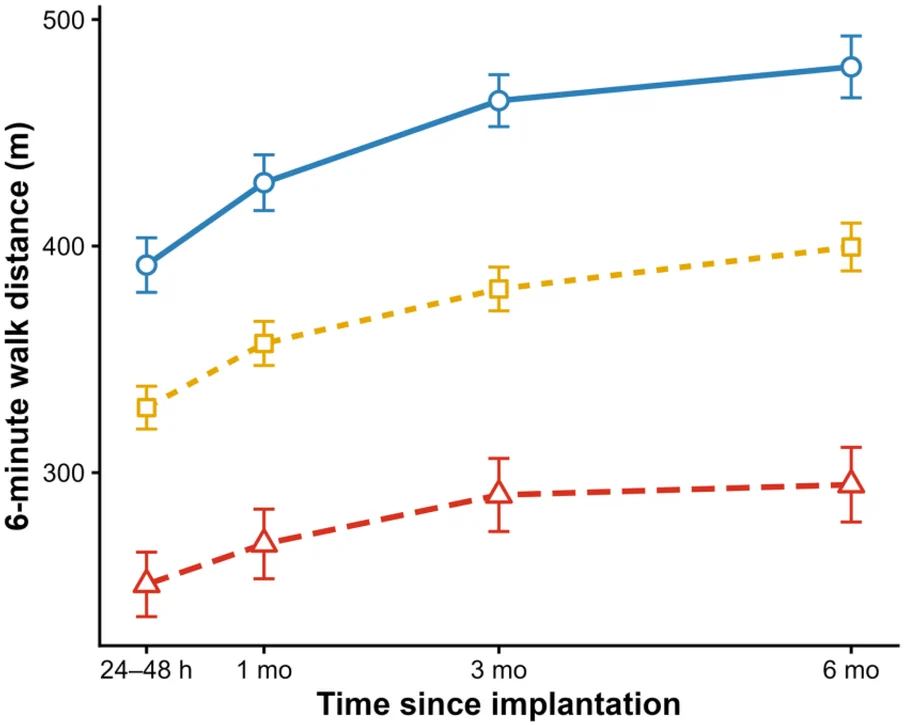
Trajectories of six-minute walk distance by kinesiophobia class. Mean six-minute walk distance in metres with 95% confidence interval at each assessment for the same three trajectory classes (colours and symbols as in Figure 1). Functional capacity improved in all groups but stayed stratified by kinesiophobia trajectory, with the persistent group walking least at every time point.

### 3.4 Baseline characteristics across trajectories

Baseline characteristics differed markedly across the three trajectories (Table 2). Compared with the low group, patients in the persistent group were older (78.0 versus 64.6 years) and had worse cardiac status, with lower ejection fraction (54.6% versus 60.0%), higher natriuretic peptide (median 265 versus 72 pg/mL), and a greater proportion in New York Heart Association class III or IV (57.1% versus 8.7%; all P below 0.01). They also had lower haemoglobin (121.5 versus 138.7 g/L) and lower estimated glomerular filtration rate (74.6 versus 84.5 mL/min/1.73 m^2^), and a heavier symptom burden. Their psychological profile was substantially worse, with higher anxiety (12.2 versus 5.5) and depression (9.8 versus 4.0), and lower exercise self-efficacy (37.1 versus 63.6) and perceived social support (54.6 versus 68.8; all P below 0.001). The persistent group also had a much shorter baseline walk distance (250.7 versus 391.6 m) and far lower rates of having received exercise guidance (31.0% versus 80.4%) and of regular exercise (16.7% versus 39.1%). By contrast, sex, body mass index, education, income, living alone, device type, hypertension, and diabetes did not differ significantly across trajectories.

**Table 2:** Baseline characteristics by kinesiophobia trajectory.

Characteristic	Low (declining) (*n* = 46)	Moderate (declining) (*n* = 72)	High (persistent) (*n* = 42)	*P*
Demographics				
Age, years	64.6 ± 14.2	72.5 ± 9.6	78.0 ± 10.2	<0.001
Female sex, *n* (%)	27 (58.7)	33 (45.8)	22 (52.4)	0.389
Body mass index, kg/m^2^	23.7 ± 3.3	24.4 ± 3.1	24.0 ± 2.8	0.569
Living alone, *n* (%)	4 (8.7)	9 (12.5)	6 (14.3)	0.733
Cardiac status				
LVEF, %	60.0 ± 7.4	57.2 ± 8.1	54.6 ± 8.1	0.007
Left atrial diameter, mm	34.4 ± 3.5	35.6 ± 3.9	36.4 ± 4.5	0.061
NYHA class III or IV, *n* (%)	4 (8.7)	14 (19.4)	24 (57.1)	<0.001
BNP, pg/mL, median (IQR)	72 (43 to 104)	136 (99 to 213)	265 (184 to 440)	<0.001
Hypertension, *n* (%)	10 (21.7)	31 (43.1)	16 (38.1)	0.058
Diabetes mellitus, *n* (%)	6 (13.0)	20 (27.8)	5 (11.9)	0.051
Dual-chamber device, *n* (%)	40 (87.0)	66 (91.7)	39 (92.9)	0.526
Laboratory				
Haemoglobin, g/L	138.7 ± 9.5	132.0 ± 10.4	121.5 ± 10.4	<0.001
eGFR, mL/min/1.73 m^2^	84.5 ± 14.4	79.1 ± 12.1	74.6 ± 13.1	0.002
Symptom and psychological status				
Symptom-burden score	0.5 ± 0.6	1.3 ± 1.1	2.0 ± 1.1	<0.001
Anxiety (HADS-A)	5.5 ± 2.8	8.1 ± 2.8	12.2 ± 2.5	<0.001
Depression (HADS-D)	4.0 ± 2.2	6.4 ± 2.3	9.8 ± 2.8	<0.001
Exercise self-efficacy (SEE)	63.6 ± 12.4	51.7 ± 10.1	37.1 ± 11.7	<0.001
Perceived social support (MSPSS)	68.8 ± 8.1	62.0 ± 9.3	54.6 ± 10.0	<0.001
Functional capacity and rehabilitation				
Baseline 6MWD, m	391.6 ± 41.6	328.7 ± 40.9	250.7 ± 47.1	<0.001
Received exercise guidance, *n* (%)	37 (80.4)	40 (55.6)	13 (31.0)	<0.001
Regular exercise habit, *n* (%)	18 (39.1)	30 (41.7)	7 (16.7)	0.018

Values are mean ± SD, median (IQR), or n (%). *P* values are from one-way ANOVA, the Kruskal–Wallis test, or the chi-square or Fisher exact test. LVEF, left ventricular ejection fraction; NYHA, New York Heart Association; BNP, B-type natriuretic peptide; IQR, interquartile range; eGFR, estimated glomerular filtration rate; HADS, hospital anxiety and depression scale; SEE, self-efficacy for exercise; MSPSS, multidimensional scale of perceived social support; 6MWD, six-minute walk distance.

### 3.5 Independent predictors of trajectory membership

In the multinomial logistic regression, with the low group as the reference, several baseline factors independently predicted an unfavourable trajectory (Table 3; Figure 3). For membership of the persistent group, the strongest psychological predictors were depression (odds ratio 2.26 per point, 95% confidence interval 1.47 to 3.50) and anxiety (1.68, 1.11 to 2.53), whereas greater exercise self-efficacy was protective (0.89, 0.80 to 0.99). Better baseline functional capacity was also protective (walk distance 0.55 per 10 m, 0.42 to 0.73). The largest effect in the model was for exercise guidance: having received it was strongly protective against a persistent course (0.04, 0.003 to 0.39). Lower haemoglobin showed a borderline association (0.35 per 10 g/L, 0.12 to 1.02), whereas age and ejection fraction were no longer significant once psychological and functional factors were taken into account. For the moderate group, depression (1.60, 1.20 to 2.14), a shorter baseline walk distance (0.78 per 10 m, 0.66 to 0.93), and lower perceived social support (0.93, 0.87 to 1.00) were independent predictors. Variables that had differed in the unadjusted comparison, including age, ejection fraction, and natriuretic peptide, were no longer independent once distress, self-efficacy, and baseline capacity were included, which indicates that their influence on the trajectory was largely carried by these more proximal factors. The pattern for the moderate group was a milder version of that for the persistent group, consistent with a graded relationship between baseline risk and the eventual course of fear.

**Table 3:** Multinomial logistic regression: baseline predictors of kinesiophobia trajectory membership.

Predictor (per unit increase)	Moderate vs. low, OR (95% CI)	*P*	High vs. low, OR (95% CI)	*P*
Age (per 5 years)	1.11 (0.83 to 1.48)	0.493	0.69 (0.39 to 1.24)	0.213
LVEF (per 5%)	1.11 (0.74 to 1.65)	0.617	0.98 (0.54 to 1.77)	0.937
Haemoglobin (per 10 g/L)	0.87 (0.47 to 1.61)	0.661	0.35 (0.12 to 1.02)	0.055
Baseline 6MWD (per 10 m)	0.78 (0.66 to 0.93)	0.005	0.55 (0.42 to 0.73)	<0.001
Anxiety, HADS-A (per 1)	1.10 (0.86 to 1.40)	0.464	1.68 (1.11 to 2.53)	0.013
Depression, HADS-D (per 1)	1.60 (1.20 to 2.14)	0.001	2.26 (1.47 to 3.50)	<0.001
Exercise self-efficacy (per 1)	0.96 (0.90 to 1.02)	0.191	0.89 (0.80 to 0.99)	0.027
Perceived social support (per 1)	0.93 (0.87 to 1.00)	0.047	1.01 (0.91 to 1.13)	0.855
Exercise guidance (yes vs. no)	0.30 (0.08 to 1.19)	0.088	0.04 (0.003 to 0.39)	0.006

The low (declining) trajectory is the reference category. Odds ratios (OR) with 95% confidence intervals (CI). Continuous predictors are expressed per the unit shown. LVEF, left ventricular ejection fraction; 6MWD, six-minute walk distance; HADS, hospital anxiety and depression scale.

**Figure 3:**
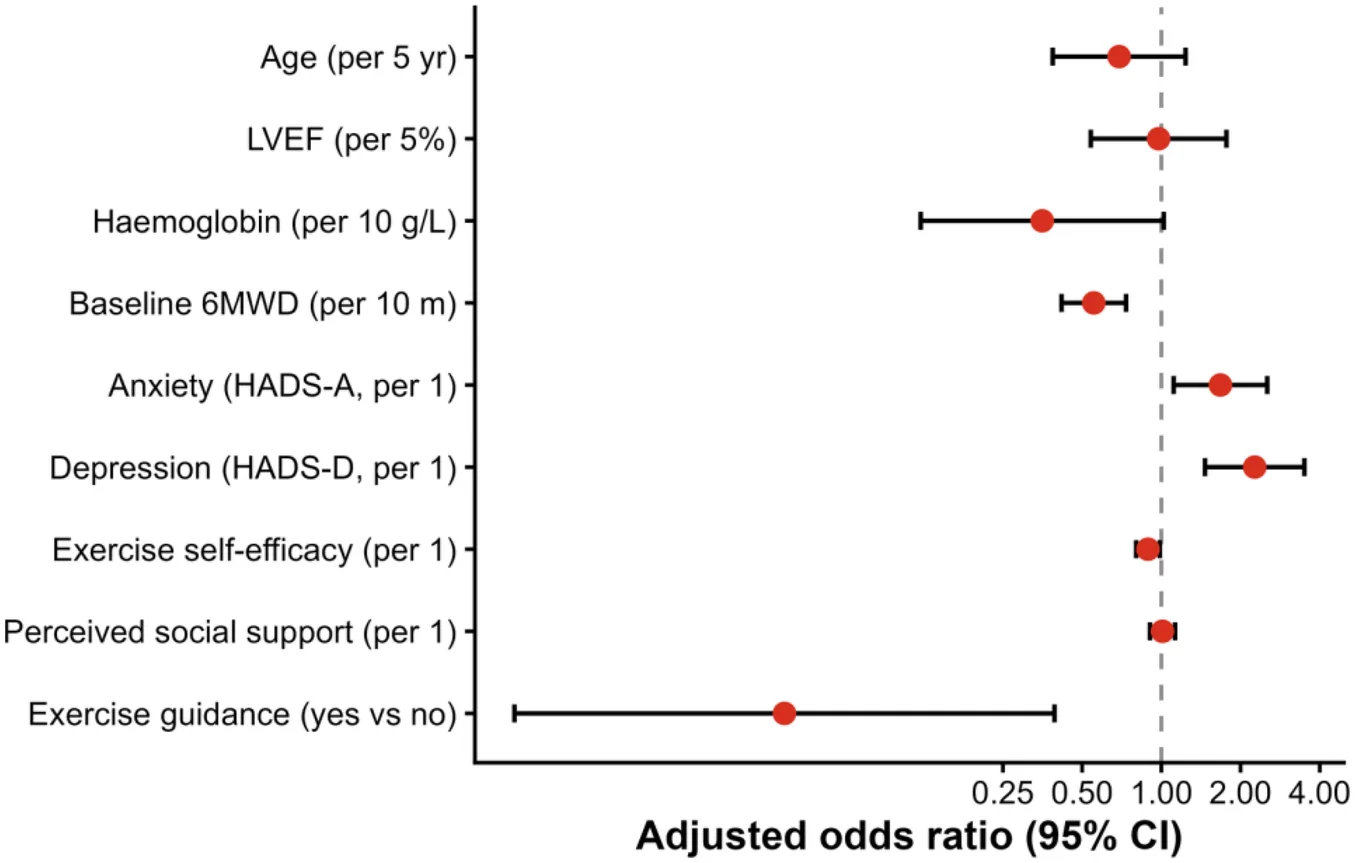
Adjusted odds ratios for membership of the high and persistent kinesiophobia trajectory. Forest plot of adjusted odds ratios with 95% confidence intervals from the multinomial logistic regression for the high and persistent versus low and declining trajectory. The dashed vertical line indicates an odds ratio of 1; values to the left indicate factors protective against a persistent course and values to the right indicate risk factors. The *x*-axis is on a logarithmic scale.

## 4 Discussion

In this cohort of patients receiving a pacemaker or defibrillator, kinesiophobia was common in the early period after implantation and, on average, declined over the following six months. Yet this average hides three distinct courses. Most patients followed a low or moderately elevated trajectory and regained confidence in movement, but about one quarter belonged to a trajectory that remained above the clinical threshold throughout follow-up. This persistently fearful subgroup is the central clinical concern, and characterising it was the main aim of the study.

The persistent trajectory was not a random collection of patients but a recognisable and vulnerable clinical phenotype. These patients were older and had objectively worse cardiac status, with lower ejection fraction, higher natriuretic peptide, and a greater proportion in advanced functional class. They were more often anaemic, had poorer renal function, and carried a heavier symptom burden. Their psychological profile was also worse, with markedly higher anxiety and depression and lower exercise self-efficacy and perceived social support. They walked the shortest distance at every assessment and gained the least over time. The close parallel between the kinesiophobia and walk-distance trajectories fits the fear-avoidance model. Persistent fear of movement and physical deconditioning appear to reinforce one another, plausibly forming a self-sustaining loop in which fear limits activity, inactivity erodes capacity, and reduced capacity then confirms the fear (4).

When all baseline factors were considered together, the independent predictors of a persistently fearful course were mainly psychological and functional rather than purely cardiac. Depression and anxiety remained the strongest predictors, and lower exercise self-efficacy was independently protective, whereas age and ejection fraction lost significance after adjustment. This pattern suggests that much of the influence of cardiac severity on fear of movement operates through psychological distress and reduced confidence. It also suggests that whether fear of movement persists depends less on the severity of the underlying cardiac condition than on the distress and low confidence that accompany it, and these are factors that can be modified. The finding is consistent with work in coronary and heart-failure patients, in whom anxiety, low self-efficacy, and depressive symptoms are repeatedly associated with higher fear of movement (31), and with the broader evidence linking psychological distress to outcomes in device recipients (12, 13).

The most actionable result concerns exercise guidance. Having received structured exercise guidance was the single strongest correlate of avoiding a persistent trajectory, and patients who received it were far less likely to remain fearful. The observational design does not allow a causal claim, and the association must be read with care, because guidance was not allocated at random and may itself have been influenced by how patients presented. Even so, its size and consistency make structured guidance a reasonable candidate for prospective testing as a simple, low-cost intervention. Reassurance built around a graded and supervised activity plan directly addresses the catastrophic beliefs that sustain fear of movement and helps build the self-efficacy that the data identify as protective. This interpretation aligns with the established benefits of exercise-based cardiac rehabilitation, which improves functional capacity, quality of life, and prognosis across a range of cardiac conditions (21, 22), and with the trial evidence and guidance that support rehabilitation specifically for defibrillator recipients (17). A reduction in fear of movement may itself be one mechanism through which rehabilitation works, since exercise-based programmes have been shown to lower kinesiophobia in coronary patients (8).

Because the persistently fearful subgroup is already distinguishable at baseline by its psychological and functional profile, a brief screening approach is feasible. A single measurement of kinesiophobia before discharge, for example with the Tampa Scale for Kinesiophobia Heart, could be combined with short assessments of anxiety, depression, and exercise self-efficacy together with a baseline walk test to stratify risk early. Patients identified as being at high risk could then be prioritised for an integrated pathway that combines psychological support with individualised and progressively dosed exercise guidance. This would move care away from uniform reassurance toward targeted and resource-efficient rehabilitation. Studies of perceived barriers and patient experience suggest that misinformation and inconsistent advice reinforce avoidance, so clear education and a consistent message from the whole clinical team are likely to be important parts of such a pathway (32, 33).

In a device clinic this pathway could be built around existing contacts rather than requiring new visits. The pre-discharge assessment offers a natural point for brief screening, and the routine checks at one and three months provide opportunities to repeat a short measure of fear and to adjust support. Patients flagged as high risk could be referred to exercise-based rehabilitation with an explicit focus on graded activity and on correcting specific fears about the device and the leads, while those at lower risk receive standard reassurance. Embedding screening in this way keeps the added burden small and uses the device follow-up service, which already sees these patients, as the platform for early intervention. The strongest modifiable factor in our data, receipt of structured exercise guidance, is exactly the kind of input that such a pathway would deliver.

Our three-class structure is consistent with cross-sectional studies that identify low, moderate, and high profiles of fear of movement in cardiac patients (18, 19), and it extends that work by showing how these levels behave over time. The overall decline we observed, with the proportion above the high-fear threshold roughly halving over six months, matches longitudinal observations in coronary patients in whom fear of movement tends to ease as recovery proceeds (34). The persistence of high fear in a substantial minority, however, mirrors the more general finding that distress after a cardiac event does not resolve in everyone and that a stable high-risk group can be identified early. A caveat is warranted here: the three trajectories differed mainly in their overall level rather than in the shape of their change, and the persistent group also improved to some extent over the six months. Its defining feature is that fear began and remained above the clinical threshold, not that it followed a qualitatively different course, and we did not formally test whether the slopes differed between the classes.

Several recent studies from cardiac populations support and complement these findings. In patients after cardiac surgery, latent profile analysis separated kinesiophobia into distinct low, moderate, and high groups, with psychological state and self-efficacy distinguishing the groups (18), which matches the baseline profile we found for the persistent trajectory. A concept analysis of kinesiophobia in coronary heart disease placed fear-provoking appraisals, emotional distress, and avoidance behaviour at the centre of the construct (19). Work in older patients with chronic heart failure, using structural equation modelling, identified self-efficacy and social support as important influences on fear of movement (11). Our results add a temporal dimension to this work. The literature has been largely cross-sectional, with longitudinal evidence only beginning to emerge: latent-class growth modelling in patients after cardiac surgery has recently identified three distinct kinesiophobia trajectories over the first months of recovery (35). Our study extends this emerging longitudinal work to recipients of a permanent pacemaker or defibrillator, a group not previously studied in this way, by showing that the high-fear profile, rather than being confined to a single moment, can persist for at least six months in a sizeable subgroup, and by linking that persistence to a concurrent deficit in measured functional capacity.

The self-reported fear was also mirrored by an objective measure of capacity. Much of the existing cardiac kinesiophobia literature relies on self-report alone (36), leaving open the possibility that high scores reflect a reporting tendency rather than a real limitation. The steady separation of the six-minute walk distance across the three trajectories, present at baseline and maintained throughout follow-up, argues against that interpretation and indicates that the fear captured by the questionnaire has a tangible functional counterpart. This strengthens the case for treating persistent kinesiophobia as a clinical target rather than as a purely subjective complaint.

This study has several strengths. The data comprised four structured assessments spanning the clinically important first six months, administered at fixed visits under a uniform protocol, and follow-up was complete because of the dedicated device service. Self-reported fear was corroborated by an objective measure of functional capacity, and a broad set of clinical, laboratory, psychological, and rehabilitation variables was analysed with trajectory modelling rather than reduced to a single mean. The high classification quality supports the validity of the identified groups.

Several limitations should be acknowledged. The study was conducted at a single centre with a moderate sample, which may limit generalisability and the precision of estimates for the smaller groups; the very wide confidence interval around the effect of exercise guidance reflects this. The design was retrospective, drawing on routinely collected follow-up records, and the study was not preregistered, so the analyses cannot be regarded as testing pre-specified hypotheses. The sample size rested on a scale-validation convention rather than a formal power calculation, and with 42 patients in the smallest class the ratio of observations to parameters in the multinomial model was modest; the predictor analysis should therefore be read as exploratory and hypothesis-generating, and the *P* values should not be given a strict confirmatory interpretation. The observational design cannot establish that exercise guidance prevents persistent kinesiophobia, and residual confounding may remain, since patients who received guidance may have differed in ways that were not measured. Reverse influence is also possible: more anxious patients may have attracted additional reassurance, while the frailest may have been advised against early activity, which would inflate the apparent protective association of guidance. The trajectory model assumed a common quadratic course within each class without random effects, and a growth mixture specification allowing individual variation around the class trajectory might partition patients somewhat differently. Exercise guidance and exercise habit were recorded as receipt and self-report rather than as a verified dose, and the content of guidance was not standardised. Follow-up ended at six months, so longer-term trajectories are unknown. The cohort was also dominated by dual-chamber pacemaker recipients; defibrillator and resynchronisation devices were few, and because these devices carry a different psychological context, the findings should not be extended to those groups without confirmation. Finally, the cohort came from a single region, and cultural factors may influence both the experience of fear and the uptake of activity. Fear of movement was measured by self-report, and although it was corroborated by the walk test we did not record objective daily physical activity, which would have allowed the link between fear and behaviour to be examined directly. The instruments are validated but were applied in a single language and setting, so their measurement properties in other populations cannot be assumed.

The logical next steps are multicentre studies to confirm the trajectory structure and its predictors, and randomised trials that test structured exercise-guidance pathways, with or without added psychological support, in patients identified at high risk. Such trials should measure fear of movement, functional capacity, physical activity, and clinical outcomes together, so that the mechanisms that link rehabilitation, fear, and recovery can be clarified.

## 5 Conclusions

Kinesiophobia after implantation of a cardiac pacemaker or defibrillator does not resolve uniformly. It follows three distinct trajectories, and about one quarter of patients remain persistently fearful and functionally limited over the first six months. This group can be identified at baseline by greater anxiety and depression, lower exercise self-efficacy, poorer functional capacity, and, most consistently, the absence of structured exercise guidance. Early screening for fear of movement, combined with targeted and exercise-based rehabilitation for patients at high risk, offers a practical way to interrupt the cycle of fear and deconditioning and to support recovery after device implantation. Because the highest-risk patients can be recognised before discharge and the device clinic already provides regular contact, the main elements of such an approach could be put in place without major additional resources.

## Data Availability

The original contributions presented in the study are included in the article/Supplementary Material, further inquiries can be directed to the corresponding author.

## References

[1] Glikson M Nielsen JC Kronborg MB Michowitz Y Auricchio A Barbash IM. 2021 ESC guidelines on cardiac pacing and cardiac resynchronization therapy. Eur Heart J. (2021) 42(35):3427–520. 10.1093/eurheartj/ehab364 34455430

[2] Bull FC Al-Ansari SS Biddle S Borodulin K Buman MP Cardon G. World health organization 2020 guidelines on physical activity and sedentary behaviour. Br J Sports Med. (2020) 54(24):1451–62. 10.1136/bjsports-2020-102955 33239350 PMC7719906

[3] Kori SH Miller RP Todd DD. Kinesiophobia: a new view of chronic pain behaviour. Pain Manag. (1990) 3:35–43.

[4] Vlaeyen JWS Linton SJ. Fear-avoidance and its consequences in chronic musculoskeletal pain: a state of the art. Pain. (2000) 85(3):317–32. 10.1016/S0304-3959(99)00242-0 10781906

[5] Bäck M Cider Å Herlitz J Lundberg M Jansson B. The impact on kinesiophobia (fear of movement) by clinical variables for patients with coronary artery disease. Int J Cardiol. (2013) 167(2):391–7. 10.1016/j.ijcard.2011.12.107 22305808

[6] Bäck M Jansson B Cider Å Herlitz J Lundberg M. Validation of a questionnaire to detect kinesiophobia (fear of movement) in patients with coronary artery disease. J Rehabil Med. (2012) 44(4):363–9. 10.2340/16501977-0942 22366980

[7] Brunetti ND Guerra A Ieva R Correale M Santoro F Tarantino N. Scared for the scar: fearsome impact of acute cardiovascular disease on perceived kinesiophobia (fear of movement). Clin Cardiol. (2017) 40(7):480–4. 10.1002/clc.22682 28272813 PMC6490519

[8] Bäck M Cider Å Herlitz J Lundberg M Jansson B. Kinesiophobia mediates the influences on attendance at exercise-based cardiac rehabilitation in patients with coronary artery disease. Physiother Theory Pract. (2016) 32(7):571–80. 10.1080/09593985.2016.1229828 27726471

[9] Keessen P Kan KJ Ter Riet G Visser B Jørstad H Latour C. Impact of kinesiophobia on initiation of cardiac rehabilitation: a prospective cohort path analysis. BMJ Open. 2022 12(11):e066435. 10.1136/bmjopen-2022-066435 36428018 PMC9703333

[10] Knapik A Dąbek J Brzęk A. Kinesiophobia as a problem in adherence to physical activity recommendations in elderly Polish patients with coronary artery disease. Patient Prefer Adherence. (2019) 13:2129–35. 10.2147/PPA.S216196 31908420 PMC6924585

[11] Qin J Xiong J Chen C Wang X Gao Y Zhou Y. Influencing factors of kinesiophobia in older patients with chronic heart failure: a structural equation model. Clin Cardiol. (2023) 46(7):729–36. 10.1002/clc.24024 37114367 PMC10352966

[12] Magyar-Russell G Thombs BD Cai JX Baveja T Kuhl EA Singh PP. The prevalence of anxiety and depression in adults with implantable cardioverter defibrillators: a systematic review. J Psychosom Res. (2011) 71(4):223–31. 10.1016/j.jpsychores.2011.02.014 21911099

[13] Ghezzi ES Sharman RLS Selvanayagam JB Psaltis PJ Sanders P Astley JM. Burden of mood symptoms and disorders in implantable cardioverter defibrillator patients: a systematic review and meta-analysis of 39 954 patients. Europace. (2023) 25(6):euad130. 10.1093/europace/euad130 37311667 PMC10264222

[14] Sears S Hauf J Kirian K Hazelton A Conti J. Posttraumatic stress and the implantable cardioverter-defibrillator patient: what the electrophysiologist needs to know. Circ Arrhythm Electrophysiol. (2011) 4(2):242–50. 10.1161/CIRCEP.110.957670 21505176

[15] Krauze N Smolis-Bąk E Kowalik I Sterliński M. Patient-reported quality of life, depression, anxiety, and physical activity in patients receiving an implantable cardioverter-defibrillator for primary versus secondary prevention: a single-centre, prospective, observational cohort study. Int J Environ Res Public Health. (2022) 19(19):12830. 10.3390/ijerph191912830 36232129 PMC9564861

[16] Ambrosetti M Abreu A Corrà U Davos CH Hansen D Frederix I. Secondary prevention through comprehensive cardiovascular rehabilitation: from knowledge to implementation. 2020 update. A position paper from the secondary prevention and rehabilitation section of the European association of preventive cardiology. Eur J Prev Cardiol. (2021) 28(5):460–95. 10.1177/2047487320913379 33611446

[17] Nielsen KM Zwisler AD Taylor RS Svendsen JH Lindschou J Anderson L. Exercise-based cardiac rehabilitation for adult patients with an implantable cardioverter defibrillator. Cochrane Database Syst Rev. (2019) 2(2):CD011828. 10.1002/14651858.CD011828.pub2 30746679 PMC6953352

[18] Xing W Piao JJ Ren T Liang Y Li Q Gu Y. Classification of kinesiophobia in patients after cardiac surgery under extracorporeal circulation in China: latent profile and influencing factors analysis from a cross-sectional study. BMJ Open. (2025) 15(1):e083909. 10.1136/bmjopen-2024-083909 39855652 PMC11758704

[19] Zhang MJ Liu S Xiong XY Liu MD Xiang Q. Kinesiophobia in patients with coronary heart disease: a Rodgers’ evolutionary concept analysis. Front Psychol. (2025) 16:1499962. 10.3389/fpsyg.2025.1499962 40207110 PMC11978840

[20] Nagin DS Odgers CL. Group-based trajectory modeling in clinical research. Annu Rev Clin Psychol. (2010) 6:109–38. 10.1146/annurev.clinpsy.121208.131413 20192788

[21] Dibben GO Faulkner J Oldridge N Rees K Thompson DR Zwisler A-D. Exercise-based cardiac rehabilitation for coronary heart disease: a meta-analysis. Eur Heart J. (2023) 44(6):452–69. 10.1093/eurheartj/ehac747 36746187 PMC9902155

[22] Taylor RS Dalal HM McDonagh STJ. The role of cardiac rehabilitation in improving cardiovascular outcomes. Nat Rev Cardiol. (2022) 19(3):180–94. 10.1038/s41569-021-00611-7 34531576 PMC8445013

[23] Anthoine E Moret L Regnault A Sébille V Hardouin JB. Sample size used to validate a scale: a review of publications on newly-developed patient reported outcomes measures. Health Qual Life Outcomes. (2014) 12:176. 10.1186/s12955-014-0176-2 25492701 PMC4275948

[24] Zigmond AS Snaith RP. The hospital anxiety and depression scale. Acta Psychiatr Scand. (1983) 67(6):361–70. 10.1111/j.1600-0447.1983.tb09716.x 6880820

[25] Bjelland I Dahl AA Haug TT Neckelmann D. The validity of the hospital anxiety and depression scale: an updated literature review. J Psychosom Res. (2002) 52(2):69–77. 10.1016/S0022-3999(01)00296-3 11832252

[26] Resnick B Jenkins LS. Testing the reliability and validity of the self-efficacy for exercise scale. Nurs Res. (2000) 49(3):154–9. 10.1097/00006199-200005000-00007 10882320

[27] Bandura A. Self-efficacy: toward a unifying theory of behavioral change. Psychol Rev. (1977) 84(2):191–215. 10.1037/0033-295X.84.2.191 847061

[28] Zimet GD Dahlem NW Zimet SG Farley GK. The multidimensional scale of perceived social support. J Pers Assess. (1988) 52(1):30–41. 10.1207/s15327752jpa5201_2 2280326

[29] ATS Committee on Proficiency Standards for Clinical Pulmonary Function Laboratories. ATS statement: guidelines for the six-minute walk test. Am J Respir Crit Care Med. (2002) 166(1):111–7. 10.1164/ajrccm.166.1.at1102 12091180

[30] Proust-Lima C Philipps V Liquet B. Estimation of extended mixed models using latent classes and latent processes: the R package lcmm. J Stat Softw. (2017) 78(2):1–56. 10.18637/jss.v078.i02

[31] Li L Piao JJ Shang Z. Fatigue and physical activity in post-CABG patients: the parallel mediation model of kinesiophobia and self-efficacy: a prospective multicentre and cross-sectional study in China. BMJ Open. (2024) 14(6):e082576. 10.1136/bmjopen-2023-082576 38951011 PMC11328647

[32] Zhou Y Gao X Xu J Ding X Yuan J Du S. Network analysis of perception of exercise benefits and barriers and kinesiophobia among patients with cardiovascular diseases. Heart Lung. (2024) 64:182–8. 10.1016/j.hrtlng.2023.12.006 38281371

[33] Bäck M Caldenius V Svensson L Lundberg M. Perceptions of kinesiophobia in relation to physical activity and exercise after myocardial infarction: a qualitative study. Phys Ther. (2020) 100(12):2110–9. 10.1093/ptj/pzaa159 32886775

[34] Bäck M Lundberg M Cider Å Herlitz J Jansson B. Relevance of kinesiophobia in relation to changes over time among patients after an acute coronary artery disease event. J Cardiopulm Rehabil Prev. (2018) 38(4):224–30. 10.1097/HCR.0000000000000265 28671936

[35] Chen Y Guo H Peng Y Niu Q Jiang Y Luo B. The latent class and influencing factors of kinesiophobia in patients after cardiac surgery with cardiopulmonary bypass: a prospective longitudinal study. J Psychosom Res. (2025) 196:112310. 10.1016/j.jpsychores.2025.112310 40644738

[36] Zeng Z Shen Y Wan L Yang X Hu Q Luo H. Kinesiophobia in patients after cardiac surgery: a scoping review. BMC Cardiovasc Disord. (2024) 24(1):469. 10.1186/s12872-024-04140-2 39223455 PMC11370225

